# Radiographic and Patient-Reported Outcomes of Lordotic Versus Non-lordotic Static Interbody Devices in Minimally Invasive Transforaminal Lumbar Interbody Fusion: A Longitudinal Comparative Cohort Study

**DOI:** 10.7759/cureus.21273

**Published:** 2022-01-15

**Authors:** Michael H Lawless, Chad F Claus, Doris Tong, Noah Jordan, Amarpal Dosanjh, Connor T Hanson, Daniel A Carr, Clifford M Houseman

**Affiliations:** 1 Division of Neurosurgery, Ascension Providence Hospital, Michigan State University, College of Human Medicine, Southfield, USA; 2 Surgery, University of Kentucky College of Medicine, Lexington, USA; 3 College of Osteopathic Medicine, Michigan State University, East Lansing, USA

**Keywords:** tlif, transforaminal lumbar interbody fusion, lordotic interbody device, lordotic cage, minimally invasive, lumbar osteoarthritis

## Abstract

Introduction

Minimally invasive transforaminal lumbar interbody fusion (MI-TLIF) is increasingly used to treat lumbar degenerative pathology. Its effect on sagittal parameters remains controversial. Static and expandable lordotic interbody devices (cages) were developed to improve segmental and overall lumbar lordosis. This study aimed to compare the radiographic and patient-reported outcomes (PROs) between static lordotic and non-lordotic titanium cages in patients undergoing 1-2 level MI-TLIF for degenerative conditions.

Methods

We reviewed consecutive eligible patients who underwent 1-2 level MI-TLIF (7/2017-11/2019) at a single institution by multiple surgeons. Standing X-rays and PROs were collected at preoperative, 1-month, and 6-month postoperative intervals. Using univariate analyses, we compared the two cohorts regarding confounders, radiographic parameters, and proportions of patients reaching minimal clinically important difference (MCID) for PROs.

Results

One-hundred-twenty-five patients were reviewed. Forty-seven had lordotic and seventy-eight non-lordotic cages. The lordotic cohort was significantly younger than the non-lordotic (55.9 years vs. 60.7 years, p= 0.042). The baseline radiographic parameters were not significantly different between cohorts. At the preoperative-6-month interval, the lordotic cohort had significant improvement in lumbar lordosis versus non-lordotic cohort (2.95° ± 7.2° vs. -0.3° ± 7.1°, p=0.024). Both cohorts showed improvement in segmental lordosis, anterior and posterior interspace height, and low subsidence grade with no significant difference between cohorts at all intervals. Overall, 69.1-83.8% of patients achieved MCID in all PROs with no significant difference between cohorts.

Conclusions

The use of a static lordotic titanium cage in 1-2 level MI-TLIF did not result in significantly different radiographic improvements or PROs compared with a non-lordotic cage.

## Introduction

Minimally invasive transforaminal lumbar interbody fusion (MI-TLIF) has become a popular and effective technique for the surgical management of focal lumbar degenerative pathology due to shorter hospital stays, quicker return to work, and increased cost-effectiveness compared to a traditional open posterior lumbar interbody fusion [1-3]. Literature supports the maintenance of spinopelvic harmony (pelvic incidence-lumbar lordosis mismatch within 9°) for improved clinical outcomes [4]. There is controversy regarding the effect of MI-TLIF on sagittal parameters due to its indeterminate effect on the lengthening of the anterior spinal column [5-7]. 

Multiple biomedical device companies have developed static and expandable lordotic interbody devices (cages) to improve segmental and regional lordosis to combat sagittal imbalance. However, the actual effect of devices remains unproven. This study compared the radiographic and clinical outcomes in patients undergoing 1-2 level MI-TLIF with static lordotic to non-lordotic titanium cages.

We hypothesize that a lordotic cage will lead to a more significant increase in segmental and lumbar lordosis compared to a non-lordotic cage.

## Materials and methods

Institutional Review Board (IRB) approval was obtained for the acquisition and analysis of this data. Due to the retrospective nature of this study, consent was not required by IRB. Our study was prepared per the Strengthening the reporting of observational studies in epidemiology (STROBE) guidelines, circa 2014 [8].

We performed a single-center retrospective review on consecutive patients who underwent one or two-level MI-TLIF between July 2017 and November 2019 using lordotic and non-lordotic titanium cages. The lordotic cages’ lordosis was 6° or 12°. The degree of lordosis was based on surgeon preference. Both lordotic and non-lordotic cages were non-articulating and from the same manufacturer (Stryker, Kalamazoo, MI). Exclusion criteria included patients undergoing revision surgery, history of prior lumbar fusion, non-degenerative indication, worker's compensation payer status, or automobile accident-related. 

Operative technique

Patients were placed under general anesthesia, intubated, and rolled prone onto a radiolucent Jackson table (Mizuho, Union City, CA). Patients were prepped and draped in a standard sterile fashion. Bilateral pedicles at operative levels were cannulated with a Jamshidi needle, and Kirshner wires were placed under biplanar fluoroscopic guidance. Fixed tubular dilators were then sequentially dilated on the ipsilateral facet to the patient’s radicular pain and secured to a rigid arm attachment (Medtronic, Minneapolis, MN). A high-speed burr followed by a combination of micro-curettes and Kerrison rongeur were then used to perform a complete facetectomy and partial laminectomy to expose Kambin’s triangle [9]. A thorough discectomy and endplate preparation was completed using a combination of curettes, disc shavers, and pituitary rongeur. Blunt trial distractors were then used to help select an appropriately sized cage. Cancellous allograft was then packed anteriorly into the disc space followed by insertion of the static titanium cage (Stryker, Kalamazoo, MI) anteriorly to engage the apophyseal ring under fluoroscopic guidance. A minimally effective dose of bone morphogenetic protein (BMP), at 1.28 mg, was then placed posterior to the cage, followed by morselized autograft obtained during facetectomy [10,11]. After confirming hemostasis, the tubular dilator was removed, and screws were inserted over guidewires and secured with rods and locking caps. The wound was closed in anatomical layers.

Clinical and radiographic measurements

Patient demographics collected included age, sex, and body mass index. For all patients, standing lateral radiographs were obtained preoperatively, at 4 weeks, and 6 months postoperatively. Patient-reported outcomes (PROs) consisted of visual analog scale (VAS) scores, Oswestry Disability Index (ODI), and the 12-item short-form health survey physical and mental component summary (SF-12 PCS, SF-12 MCS) [12-14]. PROs were collected preoperatively, 6 months, and 1 year postoperatively. Primary outcomes included clinical and radiographic outcome measures. 

**Figure 1:**
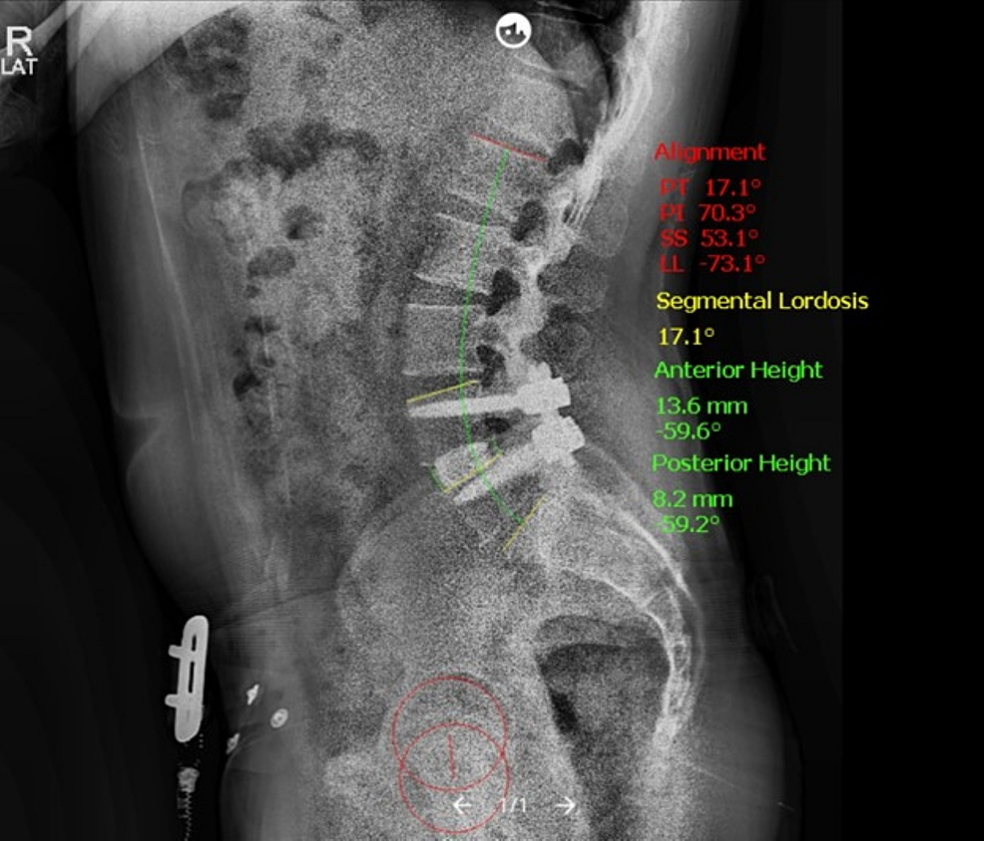
Illustrative Post-Operative Standing Radiograph Example of 6-month postoperative standing lateral radiograph of one-level lordotic cage demonstrating anterior cage placement and measured sagittal parameters using validated imaging software (Surgimap, NYC, NY).

Standing films were evaluated, and sagittal parameters were measured by neurosurgical resident physicians using validated image analysis software (Surgimap, NYC, NY) (Figure 1). Primary radiographic outcome measures included pelvic incidence (PI), pelvic tilt (PT), sacral slope (SS), lumbar lordosis (LL), PI-LL mismatch, segmental lordosis, anterior and posterior interspace height, and cage subsidence. Cage subsidence into the vertebral endplates was graded using a validated scale, with grade 0: 0%-24%, grade I: 25%-49%, grade II: 50%-74%, and grade III: 75%-100% collapse of the level [5]. 

Statistical analysis

Using previously reported differences in segmental lordosis between static and expandable lordotic cages at 17.3°± 5.4 vs. 20.3° for a power of 80%, a sample size of 102 was calculated [15]. Baseline and demographic characteristics were summarized using descriptive statistics. Paired t-tests were used to compare pre-and postoperative radiographic measures and PROs in the lordotic and non-lordotic cohorts. Minimum clinically important difference (MCID) was calculated for improvement in PRO from preoperative to 6-month and 12-month postoperative intervals between lordotic and non-lordotic cohorts [16]. For patients with two-level procedures, the interspace with the largest pre- to postoperative change was used in analyses. Sensitivity analysis was not used. Statistical significance was set at p < 0.05. Sampling bias was mitigated by using consecutive patients. Relevant confounding variables for PROs were used, including BMI, age, operative levels, sex, index PRO score, and preoperative PRO scores were used in multivariable analyses of the effect of radiographic parameters on PROs to avoid omitted variable bias. 

## Results

Patient demographics

A total of 176 patients met the inclusion criteria. Ten patients were excluded due to worker’s compensation status or injury secondary to an automobile accident and 41 patients were excluded due to prior lumbar fusion. A total of 125 patients were reviewed. All patients had a degenerative indication for surgery (e.g. spondylolisthesis, recurrent disc herniation). A total of 78 patients had a non-lordotic cage, of which 56 were one-level, and 22 were two-level. Forty-seven patients had a lordotic cage, 44 had a 6° cage, and three had a 12° cage. Thirty-nine of the lordotic cages were one-level, and eight were two-level. The lordotic cohort was younger than the non-lordotic (55.9 years vs. 60.7 years, p = 0.042) (Table 1). There was no significant difference in gender or weight distribution between cohorts (Table 1). In both the lordotic and non-lordotic cohorts, the most common operative levels were L4-5 for one-level surgery and L3-5 for two-level surgery, with no significant difference in the distribution of operative levels between both cohorts (Table 1). 

**Table 1:** Patient Demographics P-value < 0.05 considered significant; BMI, body mass index. Continuous data are reported as mean ± SD. Categorical data are reported as n (%).

N=125	Non-Lordotic (n=78)	Lordotic (n=47)	p-value
Demographic Data			
Age	60.7 ± 12.2	56.0 ± 13.1	.049
Sex			.457
Male	37 (47.4)	25 (54.3)	---
Female	41 (52.6)	21 (44.7)	---
BMI	32.6 ± 7.1	30.6 ± 5.3	.077
Number of Operative Levels			.174
1	56 (71.8)	38 (82.6)	---
2	22 (28.2)	8 (17.0)	---
Single-Level Surgeries			
Anatomical Levels			.289
L1 - L2	0	1 (2.6)	---
L2 - L3	0	0	---
L3 - L4	6 (10.7)	1 (2.6)	---
L4 - L5	33 (58.9)	22 (57.9)	---
L5 - S1	17 (30.4)	14 (36.8)	---
Two-level Surgeries			
Anatomical Levels			.875
L1 - L3	1 (4.5)	0	---
L2 - L4	1 (4.5)	0	---
L3 - L5	11 (50.0)	4 (57.1)	---
L4 - S1	9 (40.9)	3 (42.9)	---

One patient in the lordotic cohort developed an epidural hematoma requiring evacuation on postoperative day 2. Another patient in the lordotic cohort had a durotomy that required repair with dural graft, fibrin glue, and several days of lumbar catheter drainage with no subsequent cerebrospinal fluid (CSF) leak. 

Of the included patients, one patient in the non-lordotic cohort did not have a 4-week follow-up standing X-ray. Fifteen patients did not have a 6-month follow-up standing X-ray (11 - non-lordotic; 4 - lordotic).

Radiographic parameters

Non-lordotic and lordotic cohorts had similar preoperative radiographic parameter measurements, with no significantly different parameters (Table 2). The average preoperative LL was 52.1° and 50.5°, segmental lordosis 13.7° and 13.2°, PI - LL mismatch 5.3° and 1.6°, PT 20.8° and 18.7°, anterior disc height 10.2 mm and 11.1 mm, in non-lordotic and lordotic cohorts, respectively (Table 2). 

**Table 2:** Radiographic Parameters P-value < 0.05 considered significant; PI, pelvic incidence; LL, lumbar lordosis; 95% CI, 95% confidence interval. Continuous data are reported as mean ± SD and written in degrees. PI-LL mismatch presented as the absolute value.

N=125	Non-Lordotic (n=78)	Lordotic (n=45)	∆	95% CI	p-value
Lumbar Lordosis					
Preop	52.1 ± 14.8	50.5 ± 12.4	1.64	-3.30 - 6.58	.512
4 Week	50.4 ± 13.7	51.2 ± 12.9	-0.74	-5.64 - 4.17	.766
6 Month	51.7 ± 12.8	53.7 ± 11.7	-1.99	-6.77 - 2.80	.412
∆ Pre - 6 Month	-0.31 ± 7.13	2.95 ± 7.17	-3.27	-6.09 - -0.44	.024
PI – LL Mismatch					
Preop	5.25 ± 12.4	1.80 ± 12.8	3.45	-1.25 - 8.15	.148
4 Week	10.6 ± 8.1	9.27 ± 7.8	1.34	-1.59 - 4.26	.366
6 Month	8.94 ± 7.38	8.95 ± 7.21	-0.02	-2.88 - 2.84	.991
∆ Pre - 6 Month	5.78 ± 4.79	6.81 ± 5.13	-1.03	-3.00 - 0.94	.303
Pelvic Tilt					
Preop	20.8 ± 8.7	18.6 ± 9.8	2.19	-1.32 - 5.70	.219
4 Week	21.4 ± 8.7	20.0 ± 8.2	1.40	-1.71 - 4.51	.374
6 Month	20.2 ± 8.5	19.2 ± 7.7	1.06	-2.10 - 4.22	.506
∆ Pre - 6 Month	-0.79 ± 6.46	0.56 ± 6.58	-1.35	-3.93 - 1.23	.300
Sacral Slope					
Preop	36.2 ± 11.5	33.9 ± 10.6	2.30	-1.75 - 6.38	.262
4 Week	34.6 ± 12.2	34.4 ± 11.3	0.15	-4.18 - 4.47	.947
6 Month	33.9 ± 11.1	33.9 ± 10.1	-0.04	-4.15 - 4.08	.987
∆ Pre - 6 Month	-1.27 ± 6.26	0.05 ± 7.87	-1.32	-4.21 - 1.57	.366
Segmental Lordosis					
Preop	13.7 ± 8.8	13.3 ± 4.8	1.23	-2.06 - 2.81	.759
4 Week	15.3 ± 8.1	15.3 ± 5.3	-0.07	-2.49 - 2.34	.953
6 Month	14.7 ± 8.0	15.3 ± 5.3	-0.56	-3.11 - 1.99	.664
∆ Pre - 6 Month	2.27 ± 7.47	2.15 ± 6.10	0.12	-2.50 - 2.74	.926
Anterior Disc Height					
Preop	10.2 ± 4.7	11.1 ± 3.9	-0.91	-2.49 - 0.66	.254
4 Week	15.0 ± 3.4	15.4 ± 3.5	-0.34	-1.62 - 0.94	.601
6 Month	13.6 ± 3.2	14.7 ± 2.7	-1.06	-2.21 - 0.09	.069
∆ Pre - 6 Month	3.94 ± 4.28	3.56 ± 3.62	0.38	-1.15 - 1.91	.623
Posterior Disc Height					
Preop	5.62 ± 2.46	5.71 ± 1.95	-0.09	-0.89 - 0.71	.828
4 Week	8.62 ± 2.68	8.20 ± 2.71	0.42	-0.58 - 1.43	.405
6 Month	7.75 ± 2.46	7.90 ± 2.36	-0.16	-1.10 - 0.79	.744
∆ Pre - 6 Month	2.12 ± 2.68	2.10 ± 2.28	0.02	-0.94 - 0.98	.964
Cage Subsidence	---	---	---	---	.271
0	38 (56.7)	25 (59.5)	---	---	---
1	25 (37.3)	17 (40.5)	---	---	---
2	4 (6.0)	0	---	---	---

At the 4-week postoperative interval averages were as follows in non-lordotic and lordotic cohorts, respectively: LL decreased -1.4° and increased 0.7°, segmental lordosis increased 1.7° and increased 2.3°, PT increased 0.3° and increased 1.4°, PI - LL mismatch were 3.2° and 5.2°, and anterior disc height increased 5.1 mm and 4.3 mm, with no significant differences (Table 2).

The 6-month postoperative interval was as follows in non-lordotic and lordotic cohorts, respectively: LL decreased 0.3° and increased 2.9° (p = 0.023), PT decreased 0.8° and increased 0.5°, PI - LL mismatch was 8.9° and 9.0°, segmental lordosis increased 2.3° and 2.2°, and anterior disc height remained increased at 3.9 mm and 3.6 mm (Table 2). The degree of cage subsidence at 6-months postoperatively did not differ significantly between non-lordotic and lordotic cohorts, with the majority in both cohorts being grade 0 (Table 2).

Patient-reported outcomes

For PROs, 119 patients (95%) had preoperative baseline data, 81 patients (65%) had 6-month postoperative data, 70 patients (56%) had 12-month postoperative data. For all PROs, there was improvement observed at 6-month and 12-month postoperative intervals compared to preoperative baseline (Table 3).

**Table 3:** Patient-Reported Outcomes Continuous data are shown as mean ± SD. P-values < 0.05 are considered significant. VAS, visual analog scale; ODI, Oswestry disability index; SF-12, Short Form – 12 Questionnaire; PCS, physical composite score; MCS, mental composite score; MCID, minimal clinically important difference.

N=118	Non-Lordotic (n=75)	Lordotic (n=43)	% MCID Non-Lordotic	% MCID Lordotic	p-value
VAS Back					
Preop	8.29 ± 2.24	8.37 ± 2.15	---	---	---
6 Month	3.49 ± 2.71	4.36 ± 3.43	39 (86.7)	21 (60.0)	.006
12 Month	3.73 ± 3.49	2.72 ± 3.06	29 (74.4)	25 (89.3)	.128
VAS Leg					
Preop	7.27 ± 3.27	7.58 ± 3.13	---	---	---
6 Month	1.98 ± 2.90	3.06 ± 4.08	35 (76.1)	22 (62.9)	.196
12 Month	2.70 ± 3.54	1.97 ± 2.99	27 (69.2)	24 (85.7)	.119
ODI					
Preop	47.5 ± 14.4	49.6 ± 15.8	---	---	---
6 Month	28.6 ± 18.3	24.2 ± 21.4	28 (65.1)	24 (70.6)	.611
12 Month	22.6 ± 20.2	26.1 ± 21.5	21 (55.3)	17 (60.7)	.658
SF – 12 PCS					
Preop	27.6 ± 6.8	28.7 ± 7.5	---	---	---
6 Month	35.2 ± 11.1	38.5 ± 12.0	24 (53.3)	24 (70.6)	.120
12 Month	40.3 ± 11.5	39.6 ± 11.6	26 (68.4)	21 (75.0)	.560
SF – 12 MCS					
Preop	48.3 ± 11.3	45.9 ± 11.9	---	---	---
6 Month	53.2 ± 9.2	51.6 ± 10.6	22 (48.9)	17 (50.0)	.922
12 Month	54.2 ± 10.3	55.3 ± 7.7	20 (52.6)	18 (64.3)	.344

There was a significantly greater percentage of patients that met MCID in the non-lordotic versus the lordotic cohort in VAS back pain score at 6-months postoperatively (86.7% vs. 60%, p = 0.006) but not at 12-months postoperatively (74.4% vs. 89.3%, p = 0.128) (Table 3). There were no other significant differences in percentage meeting MCID in PROs between cohorts (Table 3). 

Multivariable analysis of PROs by radiographic parameters revealed pelvic tilt to be significantly associated with VAS back pain score (-0.09 β, -0.16 - -0.03 95% CI, p = 0.003) (Table 4). There were no other significant associations identified (Table 4).

**Table 4:** Multivariable Analysis of Patient-Reported Outcomes p < 0.007 considered significant; PI-LL mismatch presented as the absolute value; VAS, visual analog scale; ODI, Oswestry disability index; SF-12, Short Form – 12 Questionnaire; PCS, physical composite score; MCS, mental composite score; 95% CI, 95% confidence interval. Covariates used: Age, BMI, Operative Levels, Sex, index, preoperative PRO.

N=105	B	95% CI	p-value
VAS Back			
PI - LL Mismatch	0.05	-0.02 - 0.13	.160
Pelvic Incidence	-0.03	-0.07 - 0.01	.096
Pelvic Tilt	-0.09	-0.16 - -0.03	.003
Lumbar Lordosis	-0.02	-0.06 - 0.03	.406
Sacral Slope	0.01	-0.05 - 0.06	.826
Segmental Lordosis	-0.01	-0.11 - 0.09	.834
Anterior Disc Height	0.03	-0.20 - 0.26	.796
Posterior Disc Height	0.11	-0.16 - 0.37	.436
VAS Leg			
PI - LL Mismatch	0.002	-0.08 - 0.08	.960
Pelvic Incidence	-0.02	-0.07 - 0.02	.311
Pelvic Tilt	-0.01	-0.08 - 0.07	.857
Lumbar Lordosis	-0.01	-0.06 - 0.04	.651
Sacral Slope	-0.05	-0.11 - 0.02	.130
Segmental Lordosis	-0.05	-0.14 - 0.05	.136
Anterior Disc Height	-0.16	-0.40 - 0.07	.180
Posterior Disc Height	0.12	-0.17 - 0.41	.422
ODI			
PI - LL Mismatch	0.07	-0.31 - 0.45	.724
Pelvic Incidence	-0.14	-0.38 - 0.11	.266
Pelvic Tilt	-0.18	-0.59 - 0.24	.405
Lumbar Lordosis	-0.15	-0.41 - 0.11	.270
Sacral Slope	-0.12	-0.42 - 0.18	.424
Segmental Lordosis	-0.03	-0.75 - 0.69	.929
Anterior Disc Height	0.60	-0.69 - 1.89	.365
Posterior Disc Height	1.69	0.18 - 3.20	.028
SF - 12 PCS			
PI - LL Mismatch	-0.01	-0.26 - 0.24	.932
Pelvic Incidence	0.10	-0.05 - 0.26	.200
Pelvic Tilt	0.29	0.01 - 0.56	.042
Lumbar Lordosis	-0.02	-0.17 - 0.13	.767
Sacral Slope	0.01	-0.18 - 0.20	.924
Segmental Lordosis	-0.06	-0.37 - 0.26	.729
Anterior Disc Height	-0.16	-0.97 - 0.65	.695
Posterior Disc Height	-0.66	-1.59 - 0.27	.162
SF - 12 MCS			
PI - LL Mismatch	-0.04	-0.24 - 0.17	.715
Pelvic Incidence	0.01	-0.09 - 0.11	.861
Pelvic Tilt	0.05	-0.12 - 0.23	.539
Lumbar Lordosis	-0.04	-0.18 - 0.09	.540
Sacral Slope	-0.01	-0.14 - 0.12	.871
Segmental Lordosis	-0.05	-0.27 - 0.17	.662
Anterior Disc Height	-0.43	-1.01 - 0.16	.151
Posterior Disc Height	-0.21	-0.85 - 0.43	.523

## Discussion

This longitudinal cohort study, which included 125 patients undergoing 1- to 2-level MI-TLIF for degenerative indications with a lordotic or non-lordotic static titanium cage, revealed overall no significant differences between the radiographic outcomes or PROs between cohorts. 

There are many interbody fusion techniques to treat degenerative disc disease with mechanical instability in the lumbar spine. Transforaminal lumbar interbody fusion has remained a workhorse approach for decades since Harms’s first description in 1998 due to its effectiveness in decompression of the neural elements and stabilization of the lumbar spine [17].

With more recent innovations in minimally invasive approaches, a common criticism of the MI-TLIF approach, particularly in a unilateral approach, is that it is a kyphosing procedure [15]. To combat this, biomechanical device companies have designed a plethora of static, expandable, and articulating interbody devices to improve the segmental and overall lordosis. However, in this study, we found no clinically significant difference between the static lordotic and non-lordotic MI-TLIF titanium cages in one or two-level surgery. This could be related to the limited power of an MI-TLIF on affecting sagittal parameters. This is likely due to the lack of shortening the posterior column via a unilateral facetectomy and limited lengthening of the anterior column without the release of the anterior longitudinal ligament [5].

Key results

We did find the lumbar lordosis was increased with a static lordotic titanium cage compared to non-lordotic at 6-months postoperatively. We also demonstrated that the lordotic and non-lordotic cage did improve segmental lordosis by, on average, 2.3°. This improvement in segmental lordosis is consistent with previously reported studies [5,15]. However, these findings are discordant as one would expect a consistent effect on the segmental lordosis and lumbar lordosis. 

We did demonstrate that a lordotic cage was not associated with an increased incidence of subsidence compared with the non-lordotic cage. There was an overall low degree of subsidence in both cohorts. After radical discectomy and endplate preparation, we routinely pack 15 cubic centimeters of cancellous bone allograft into the interspace to buttress the cage to decrease excessive subsidence. We further take advantage of the increased strength of the vertebral body endplate at the midline and anterior aspect of the apophyseal ring and place the cage at this site [18,19]. By placing the cage as far anterior as the anterior perimeter of the apophyseal ring, we aim to increase the anterior interspace height and subsequently segmental and overall lordosis. Our findings demonstrate that the increase in disc height and segmental lordosis was sustained over time, which has been a criticism of static cages in prior studies [7].

Most patients did not have completely collapsed disc spaces or “vacuum phenomenon,” evidenced by the preoperative disc heights. This indicates that there was at least some maintenance of central intradiscal pressure and thus persevered bony endplate cortical structure in keeping with Wolff’s law [20]. This logical preservation of the endplate may further explain our low degree of subsidence in both cohorts. 

It is well established that pelvic tilt > 20° indicates the patient is attempting to compensate for sagittal imbalance, which would increase the workload of the paraspinal musculature, which could increase the VAS back score [4]. The average PT for lordotic and non-lordotic cohorts at the 6-month radiograph were 19.2° and 20.2°, respectively. 

Generalizability

Our results are generalizable to middle-aged patients with degenerative lumbar spine conditions undergoing one or two-level MI-TLIF with a static titanium cage. To our knowledge, this is the first study to compare the radiographic and clinical outcomes of lordotic and non-lordotic static titanium cages in MI-TLIF.

Limitations 

Due to the retrospective design, our study may be subject to some bias, particularly regarding the clinical outcome, that may not be present in a prospective study. We did not assess radiographic coronal imbalance. However, Daubs et al. demonstrated that only sagittal plane correction was a predictor of ODI improvement in the setting of combined coronal and sagittal deformity [21].

## Conclusions

This study demonstrates that the use of a static lordotic titanium cage in 1-2 level MI-TLIF did not result in significantly different radiographic or PROs compared with a non-lordotic cage. Lordotic and non-lordotic cage cohorts had low grades of subsidence and improvement in interspace height, segmental lordosis, and PROs postoperatively.
